# Comprehensive long-term efficacy and safety of recombinant human alpha-mannosidase (velmanase alfa) treatment in patients with alpha-mannosidosis

**DOI:** 10.1007/s10545-018-0175-2

**Published:** 2018-05-03

**Authors:** Allan M. Lund, Line Borgwardt, Federica Cattaneo, Diego Ardigò, Silvia Geraci, Mercedes Gil-Campos, Linda De Meirleir, Cécile Laroche, Philippe Dolhem, Duncan Cole, Anna Tylki-Szymanska, Monica Lopez-Rodriguez, Encarna Guillén-Navarro, Christine I. Dali, Bénédicte Héron, Jens Fogh, Nicole Muschol, Dawn Phillips, J. M. Hannerieke Van den Hout, Simon A. Jones, Yasmina Amraoui, Paul Harmatz, Nathalie Guffon

**Affiliations:** 1 Departments of Paediatrics and Adolescent Medicine, Centre for Inherited Metabolic Diseases, Copenhagen, Denmark; 2 Department of Clinical Genetics, Centre for Inherited Metabolic Diseases, Copenhagen University Hospital, Rigshospitalet, Copenhagen, Denmark; 3 Center for Genomic Medicine, Copenhagen University Hospital, Rigshospitalet, Copenhagen, Denmark; 4 Chiesi Farmaceutici S.p.A, Parma, Italy; 5 Unidad de Metabolismo e Investigación Pediátrica, Hospital Universitario Reina Sofía, IMIBIC, Universidad de Córdoba, CIBERObn, Córdoba, Spain; 6 Paediatric Neurology and Metabolism, Universitair Ziekenhuis, Brussels, Belgium; 7 Limoges Hospital, Limoges, France; 8 Centre Hospitalier de Saint-Quentin, Saint-Quentin, France; 9 Department of Medical Biochemistry and Immunology, University Hospital of Wales, Cardiff, Wales UK; 10 Department of Paediatric, Nutrition and Metabolic Diseases, The Children’s Memorial Health Institute, Warsaw, Poland; 11 Hospital Central Cruz Roja, Madrid, Spain; 12 Medical Genetics Section, Hospital Clínico Universitario Virgen de la Arrixaca, IMIB-Arrixaca, CIBERER-ISCIII, Madrid, Spain; 13 Service de Neuropédiatrie, Centre de Référence des Maladies Lysosomales, and Sorbonne Université, GRC n°19, pathologies Congénitales du Cervelet-LeucoDystrophies, AP-HP, Hôpital Armand Trousseau, F-75012 Paris, France; 14 Zymenex A/S, Hillerød, Denmark; 15 International Center for Lysosomal Disorders, University Medical Center Hamburg-Eppendorf, Hamburg, Germany; 16 Evidera, Bethesda, MD USA; 17 Center for Lysosomal and Metabolic Diseases (department of Pediatrics), Erasmus MC University Medical Center – Sophia Children’s Hospital, Rotterdam, The Netherlands; 18 Manchester Centre for Genomic Medicine, Central Manchester University Hospitals NHS Foundation Trust, Manchester, UK; 19 Center for Pediatric and Adolescent Medicine, Villa Metabolica, University Medical Center Mainz, Mainz, Germany; 20 UCSF Benioff Children’s Hospital Oakland, Oakland, CA USA; 21 Centre de Référence des Maladies Héréditaires du Métabolisme, Hôpital Femme Mère Enfant, Lyon, France

**Keywords:** Alpha-mannosidosis, Recombinant human alpha-mannosidase, Lysosomal storage disorder, Enzyme replacement therapy, Velmanase alfa, Integrated analysis

## Abstract

**Introduction:**

Long-term outcome data provide important insights into the clinical utility of enzyme replacement therapies. Such data are presented for velmanase alfa in the treatment of alpha-mannosidosis (AM).

**Methods:**

Patient data (*n* = 33; 14 adults, 19 paediatric) from the clinical development programme for velmanase alfa were integrated in this prospectively-designed analysis of long-term efficacy and safety. Patients who participated in the phase I/II or phase III trials and were continuing to receive treatment after completion of the trials were invited to participate in a comprehensive evaluation visit to assess long-term outcomes. Primary endpoints were changes in serum oligosaccharide and the 3-minute stair climb test (3MSCT).

**Results:**

Mean (SD) treatment exposure was 29.3 (15.2) months. Serum oligosaccharide levels were significantly reduced in the overall population at 12 months (mean change: –72.7%, *P* < 0.001) and remained statistically significant at last observation (−62.8%, *P* < 0.001). A mean improvement of +9.3% in 3MSCT was observed at 12 months (*P* = 0.013), which also remained statistically significant at last observation (+13.8%, *P* = 0.004), with a more pronounced improvement detected in the paediatric subgroup. No treatment-emergent adverse events were reported leading to permanent treatment discontinuation.

**Conclusions:**

Patients treated with velmanase alfa experienced improvements in biochemical and functional measures that were maintained for up to 4 years. Long term follow-up is important and further supports the use of velmanase alfa as an effective and well-tolerated treatment for AM. Based on the currently available data set, no baseline characteristic can be predictive of treatment outcome. Early treatment during paediatric age showed better outcome in functional endpoints.

**Electronic supplementary material:**

The online version of this article (10.1007/s10545-018-0175-2) contains supplementary material, which is available to authorized users.

## Introduction

Alpha-mannosidosis (AM) is a rare autosomal recessive lysosomal storage disorder with an estimated prevalence of one in 500,000–1,000,000 live births (Meikle et al 2004; Meikle et al 1999). Pathogenic sequence variants in the *MAN2B1* gene cause a reduction in the activity of lysosomal alpha-mannosidase, resulting in impaired glycoprotein degradation in the lysosomes, and ultimately, impaired cellular function and apoptosis (Borgwardt et al 2015; Thomas 2001). AM presents as a multi-systemic disease, characterised by immunodeficiency, hearing impairment, facial and skeletal abnormalities and mental retardation, among other manifestations (Malm and Nilssen, 2008).

Other than supportive care, the only treatment option currently available for AM is allogeneic haematopoietic stem cell transplantation (HSCT) from a human leukocyte antigens (HLA)-matched donor, which has a variable outcome and carries a serious morbidity and mortality risk (Mynarek et al 2012; Borgwardt et al 2014a).

Velmanase alfa is a recombinant human lysosomal alpha-mannosidase, developed as intravenous (IV) enzyme replacement therapy (ERT) for AM (Borgwardt et al 2013). In phase I/II trials, velmanase alfa was associated with a sustained decrease in serum oligosaccharides after 18 months of therapy (mean percentage change −89.9%, *P* < 0.001) and achievement of an average improvement of 39 steps in the 3-minute stair climb test (3MSCT; *P* = 0.004) (Borgwardt et al 2014b). Velmanase alfa treatment was subsequently evaluated in a phase III placebo-controlled randomised trial (NCT01681953; Borgwardt et al 2017 [submitted]). Here we present long-term outcomes in patients with AM treated with velmanase alfa.

## Methods

### Study design

This study is an integrated analysis of efficacy and safety outcomes in patients with AM who participated in velmanase alfa trials and received therapy for up to 4 years in follow-up clinical trial or compassionate use (CU) programme.

### Analysis population and database generation

Individual patient data from phase I/II (Borgwardt et al 2013) and III trials and the subsequent rhLAMAN-07 (NCT01908712), rhLAMAN-09 (NCT01908725) and rhLAMAN-10 (NCT02478840) studies were integrated into a single database. rhLAMAN-07 and rhLAMAN-09 are ongoing clinical trials of once-weekly 1 mg/kg velmanase alfa treatment in patients from France, or from Poland and Norway, respectively, who previously participated in velmanase alfa trials. rhLAMAN-10 is a single-centre clinical trial of 1 mg/kg velmanase alfa in which patients who had previously participated in velmanase alfa clinical trials and subsequently enrolled in the international CU programme were invited to undergo a comprehensive evaluation visit (last observation; LO). Inclusion/exclusion criteria are provided in the Supplementary methods.

Patients had a confirmed diagnosis of AM, as defined by alpha-mannosidase activity <10% of normal activity, who had participated in the phase I/II and III trials, and were currently receiving weekly IV infusions of velmanase alfa according to their respective follow-up studies or CU programmes.

### Procedures and treatment

All patients underwent clinical, functional and laboratory assessments at baseline and at pre-specified time points according to the protocol of their parental trial. Patients enrolled in rhLAMAN-07, −09 and − 10 underwent a LO visit at the same central location. In the rhLAMAN-10 trial, patients were screened for eligibility on day 1 of the visit. Patients who provided informed consent underwent pre-infusion evaluations, and were given a single IV infusion of velmanase alfa 1 mg/kg on day 2.

### Endpoints

Primary endpoints for this analysis were the change from baseline to LO in serum oligosaccharides, and change from baseline to LO in the 3MSCT. Serum oligosaccharides were measured by high pressure liquid chromatography (HPLC) with ultraviolet (UV) detection coupled with matrix-assisted laser desorption/ionisation-time of flight (MALDI-TOF) mass spectrometry. Functional capacity was further assessed using the 6-minute walk test (6MWT), forced vital capacity (FVC % predicted and l, measured by spirometry), and Bruininks-Oseretsky test of motor proficiency (BOT-2). Immunological status was assessed as serum immunoglobulin G (IgG) concentrations and presence of hypogammaglobulinaemia in patients enrolled in the phase III trial, and classified according to criteria reported in Suppl. Table 2. Level of disability during activities of daily living and health-related quality of life were assessed using the Childhood Health Assessment Questionnaire (CHAQ) and Euro QOL 5D 5 L (EQ5D5L; used in the phase III trial) surveys, respectively, and results will be presented separately. Treatment-emergent adverse events (TEAEs), adverse drug reactions (ADRs), infusion-related reactions (IRRs) and anti-drug antibodies (ADAs) were assessed throughout the trials.

### Statistical analysis

Baseline data were derived from the original trial in which patients were enrolled. LO data were derived from the last non-missing values collected per the protocol of the original trial, or long-term follow-on trial. For patients randomised to placebo in the phase III trial, the baseline for all evaluations was the last non-missing value recorded immediately prior to initiation of active treatment after study completion. Integrated analysis data were evaluated in the overall population and in age subgroups: adults (aged ≥18 years) and paediatrics (aged <18 years). The absolute changes and percentage changes from baseline to each time point were analysed for all primary and secondary efficacy endpoints, using the paired *t*-test and presented with their *P*-value and 95% CI.

## Results

### Patients

**Fig. 1:**
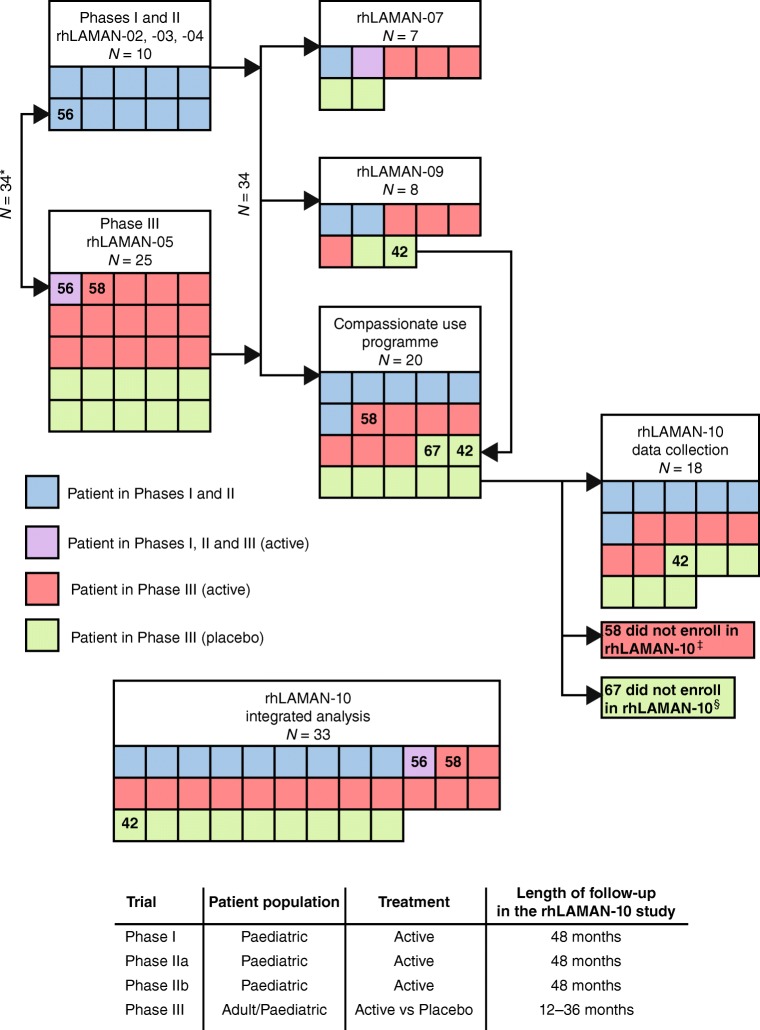
Patient disposition by parental trial and by trial/CU programme at the time of enrolment in rhLAMAN-10 (enrolment in follow-up trial or CU programme determined by national regulations). * Patient 56 participated in rhLAMAN-02 and rhLAMAN-03, discontinued treatment due to an AE but later enrolled in rhLAMAN-05. This patient is only counted once within the integrated analysis. ^‡^ Patient 58 participated in the rhLAMAN-05 study in the active arm. After completing the rhLAMAN-05 study the patient received velmanase alfa in the compassionate use programme but did not participate in the rhLAMAN-10 study. Since the subject received velmanase alfa for 12 months in the rhLAMAN-05 study, data from this patient are included in the integrated analysis. ^§^ Patient 67 participated in the rhLAMAN-05 study in the placebo arm and entered the compassionate use programme but did not participate in the rhLAMAN-10 study. As this patient did not have any data collected during active treatment, he was excluded from the integrated analysis

Thirty-four patients participated in phase I/II and III trials and received velmanase alfa treatment as part of rhLAMAN-07, −09 or CU programme. Patient disposition is shown in Fig. 1. Individual data from 33 patients were included in the integrated analysis. Fourteen patients were adult and 19 paediatric at the time of first infusion. To date, six patients have transitioned from being paediatric to adult patients during their treatment period.

Baseline demographic and functional characteristics of each patient are presented in Suppl. Table 1. All patients received the intended clinical dose of 1 mg/kg/week (equal to 25 U used in the phase I/II study; Borgwardt et al 2014b) by IV infusion for ≥12 months, and 19 (57.6%) received the intended dose for ≥24 months. Mean (standard deviation, SD) duration of exposure to treatment was 29.3 (15.2) months (range: 11.7–53.4 months). The patient population initially enrolled in the phase I/II studies (paediatric population aged 6–17 years), received velmanase alfa for up to 48 months (Fig. 1). Treatment compliance was not assessed in this study, but was reported as ≥90% in the phase I/II and III trials.

### Efficacy endpoints

**Fig. 2:**
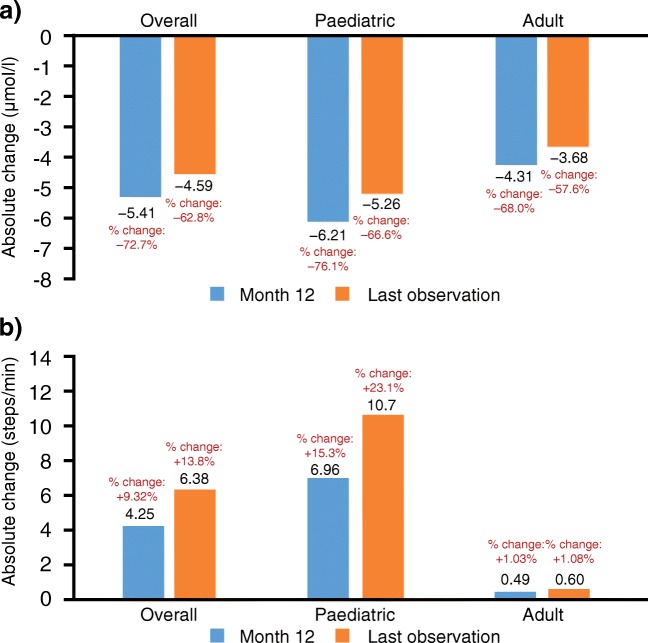
Changes from baseline in **a)** serum oligosaccharides and **b)** 3MSCT 3MSCT, 3-min stair climb test.

A statistically significant clearance of serum oligosaccharides was observed in the overall population from baseline to 12 months (*n* = 31; mean change: −72.7% [95% CI: –81.4, −64.1], *P* < 0.001) and remained statistically significant at LO (*n* = 33; mean percentage change: −62.8% [95% CI: –74.7, −50.8], *P* < 0.001). Similar results were seen across age groups (Fig. 2a and Fig. 1a in the Supplementary material). A significant improvement in 3MSCT was also observed at 12 months (*n* = 31; mean change: +9.3% [95% CI: 2.14, 16.5], *P* = 0.013) and this remained significant at LO (*n* = 33; mean change: +13.8% [95% CI: 4.61, 22.92], *P* = 0.004) (Fig. 2b). A greater improvement in 3MSCT was observed in paediatric patients, compared with adults, at both 12 months (*n* = 18; +6.96 steps/min; +15.3%) and LO (*n* = 19; +10.7 steps/min; +23.1%) (Fig. 2b and Fig. 1b in the Supplementary material). Paediatric patients with the longest treatment exposure (48 months) experienced a mean absolute increase from baseline of 17.1 steps/min (*n* = 9, +39.1%) at the end of follow-up.

A trend towards improved 6MWT was observed at 12 months and was statistically significant at LO (Table 1). Paediatric patients experienced a greater increase in the 6MWT from baseline to the LO, compared with adult patients (Table 1 and Fig. 2a in the Supplementary material. Four of five paediatric and three of the five adult patients, requiring walking help or aids (defined as cane, walker, crutches or wheelchair in CHAQ Disability Index) at baseline became independent at LO. A statistically significant mean absolute change and percentage change in FVC (percentage predicted) and FVC (L) was observed from baseline to 12 months and to LO. The absolute change in FVC (percentage predicted and L) was greater in paediatric vs adult patients at LO (Table 1 and Fig. 2b in the Supplementary material. The BOT-2 total point score for the entire group increased and the percentage change was statistically significant at LO (*P* = 0.035; Table 1 and Fig. 2c in the Supplementary material).

**Table 1:** Secondary endpoints results

Variable		Paediatric			Adults			Overall				
		N	Values *Mean (SD)*	% *Mean (SD)*	N	Values *Mean (SD)*	% *Mean (SD)*	N	Values *Mean (SD)*	95% CI *p-value*	% *Mean (SD)*	95% CI p-value
**6MWT** *(meters)*	Baseline	19	454.2 (86.3)	-	14	483.4 (95.6)	-	33	466.6 (90.1)	-	-	-
	12-month change	18	35.0 (75.7)	11.4 (29.1)	13	3.8 (43.4)	1.7 (9.8)	31	21.9 (65.2)	–2.0, 45.8 *p = 0.071*	7.3 (23.3)	–1.2, 15.9 *p = 0.090*
	Last observation change	19	39.1 (67.6)	11.9 (26.6)	14	0.3 (50.5)	0.7 (11.6)	33	22.4 (63.2)	0.0, 44.8 *p = 0.050*	7.1 (22.0)	–0.7, 14.9 *p = 0.071*
**FVC % of predicted**	Baseline	17	79.6 (16.4)	-	12	92.5 (19.4)	-	29	84.9 (18.6)	-	-	-
	12-month change	17	6.9 (14.6)	9.7 (19.3)	11	6.0 (9.9)	6.6 (11.7)	30	6.6 (12.8)	1.6, 12.5 *p = 0.011*	8.5 (16.5)	2.1, 14.9 *p = 0.011*
	Last observation change	17	11.6 (15.7)	16.4 (22)	12	3.0 (12.4)	2.1 (16.7)	31	8.1 (14.8)	2.4,13.7 *p = 0.007*	10.5 (20.9)	2.6, 18.5 *p = 0.011*
**FVC** *(liters)*	Baseline	17	2.2 (0.9)	-	12	3.2 (1.1)	-	29	2.7 (1.1)	-	-	-
	12-month change	17	0.5 (0.5)	22.1 (21.9)	11	0.2 (0.3)	7.4 (11.1)	28	0.4 (0.4)	0.27, 0.55 *p < 0.001*	16.3 (19.6)	8.7, 23.9 *p < 0.001*
	Last observation change	17	0.9 (0.7)	45.9 (39.1)	12	0.2 (0.4)	3.5 (16.3)	28	0.6 (0.7)	0.3, 0.9 *p < 0.001*	28.4 (37.8)	14.0, 42.8 *p < 0.001*
**BOT-2 Total Score**	Baseline	19	101.9 (53.8)	-	14	113.9 (38.6)	-	33	107 (47.6)	-	-	-
	12-month change	18	13.6 (17.5)	17.1 (20.6)	13	-0.9 (10.6)	1.6 (13.3)	31	7.5 (16.5)	1.4, 13.5 *p = 0.017*	10.6 (19.3)	3.5, 17.7 *p = 0.005*
	Last observation change	19	10.7 (29.5)	23 (40.1)	14	-2.5 (9.9)	-0.7 (15.9)	33	5.1 (23.9)	-3.4, 13.6 *p = 0.230*	13 (33.9)	1.0, 25.0 *p = 0,035*
**Patients Serum IgG status**		**Baseline (n=24)**			**Month 12 (n=22)**			**Last Observation (n=24)**				
	Not/slightly impaired n (%)	15 (62.5)			19 (86.4)			21 (87.5)				
	Impaired n (%)	7 (29.2)			3 (13.6)			3 (12.5)				
	Seriously Impaired n (%)	2 (8.3)			0 (0)			0 (0)				

6MWT, 6-Minute Walk Test; BOT-2; Bruininks-Oseretsky Test of Motor Proficiency; FVC, forced vital capacity; SD, standard deviation

*“Not/slightly impaired” serum IgG concentration defined relative to Cassidy et al 1974; “impaired” serum IgG concentration defined as 4 mg/mL to lower limit of normal range,

“seriously impaired serum IgG concentration defined as < 4 mg/ml

Results of additional secondary endpoints and PK analyses are reported in the Suppl. Tables 2–8. Notably, biomarker analysis did not show a significant change in cerebrospinal fluid (CSF) biomarkers from baseline to LO (Suppl. Table 6).

### Immunological status

A consistent and significant increase from baseline in mean serum IgG concentrations was observed at 12 months (*n* = 22 [data only available for patients initially enrolled in the phase III trial], mean percentage change: +47.0% [95% CI: 34.9, 59.1], *P* < 0.001) and at LO (*n* = 24; mean percentage change: +44.1% [95% CI: 32.6, 55.6], *P* < 0.001), reversing clinically relevant hypogammaglobulinaemia when present. Table 1 shows the improvements in patients’ serum immunoglobulin status with treatment.

### Safety

The incidence of treatment emergent adverse events (TEAEs) is summarised in Table 2. More ADRs were reported in paediatric patients, who had generally received more treatments. Two patients experienced serious TEAEs that were considered treatment-related: one patient experienced loss of consciousness for 2–3 min 8 days after a velmanase alfa infusion; this event resolved with no action taken regarding the study drug, and the patient was subsequently diagnosed with epilepsy; the second patient, who was receiving long-term high-dose ibuprofen therapy (600 mg/day), experienced moderate intensity acute renal failure that led to temporary discontinuation of study treatment. The renal failure resolved after 92 days. No TEAEs leading to permanent treatment discontinuation were reported.

**Table 2:** Summary of TEAEs by age and in the overall population

	Paediatric *n* = 19		Adult *n* = 14		Overall *N* = 33	
	Number of events	Number of patients (%)	Number of events	Number of patients (%)	Number of events	Number of patients (%)
Any TEAEs	423	17 (89.5)	123	12 (85.7)	546	29 (87.9)
ADRs	69	12 (63.2)	15	5 (35.7)	84	17 (51.5)
Serious TEAEs	9	7 (36.8)	5	5 (35.7)	14	12 (36.4)
Serious treatment-related TEAEs	1	1 (5.3)	1	1 (7.1)	2	2 (6.1)
Severe TEAEs	3	2 (10.5)	1	1 (7.1)	4	3 (9.1)
TEAEs with a fatal outcome	0	0 (0.0)	0	0 (0.0)	0	0 (0.0)
TEAEs leading to discontinuation	0	0 (0.0)	0	0 (0.0)	0	0 (0.0)

*ADR* adverse drug reaction, *TEAEs* treatment-emergent adverse events

Nineteen IRRs were reported in three patients (9%). Fourteen of the recorded IRRs occurred in one patient, who withdrew from the phase I/II and discontinued therapy for 21 months, but subsequently enrolled in the phase III study and is still receiving treatment. Eight patients (24.2%) developed ADAs at least once during treatment. At least one further confirmatory ADA-positive result was present in six of these patients, with ADAs levels around the cut off threshold. Two patients had an ADA titre >80 U/ml (maximum values of 1012 U/ml and 440 U/ml respectively) and experienced IRRs.

## Discussion

In this integrated analysis of the long-term efficacy of velmanase alfa treatment in patients with AM, statistically significant improvements were observed in the co-primary endpoints: serum oligosaccharide levels and 3MSCT. Secondary endpoints evaluating endurance, pulmonary function and motor proficiency also showed improvements up to 48 months, which are particularly relevant in the context of a progressively worsening disease. The long-term safety and immunogenicity profile of velmanase alfa appears compatible with chronic administration of the drug.

This study is a prospective integrated data analysis of previous clinical trials with different designs (rhLAMAN-02, −03, −04, −07 and − 09 are open-label, single-arm; rhLAMAN-05 is randomised, double-blind, parallel-group). The integrated study design was developed to address the challenges of the rarity of AM and statistical analyses of small patient populations. The study protocol was written a priori and the statistical analysis plan designed before database lock. The reason for choosing this approach arises from the rarity of the condition and the unusual possibility to be able to collect treatment data for up to 4 years before marketing authorisation.

The presence of a control group limited to the 12-month phase III trial (Borgwardt et al 2018) is, at least partially, mitigated by the duration of the follow-up and the repeated assessments.

Intra and inter-rater administrative reliability was maximised by conducting all assessments at one site with standardised administrative guidelines, and the same personnel collected the data on subsequent visits. The results of this analysis clearly support the biochemical efficacy of velmanase alfa treatment in patients with AM; marked decreases were seen in serum oligosaccharide levels, and statistically significant increases in serum immunoglobulins were observed, with correction of hypogammaglobulinaemia in many patients. Since the accumulation of mannose-rich oligosaccharides is considered the causative mechanism of cellular dysfunction and hypogammaglobulinaemia in AM, alongside with oligosaccharide accumulation in lymphocytes, and is suspected to be the cause of the increase in rate and severity of infections in AM patients (Malm et al 2000), these changes are assumed to produce a therapeutic benefit (Malm et al 2014; Muenzer 2014). The decrease in the proportion of patients who had impaired or seriously impaired immunoglobulin levels supports the use of serum immunoglobulin as an additional biomarker of velmanase alfa activity. All treated patients benefited from an improvement of IgG in serum.

Performance in functional assessments can be influenced by developmental stage, understanding of instructions and willingness to cooperate, all of which can be problematic in paediatric and/or cognitively disabled patients. These challenges, combined with the wide age range of study patients (6–35 years), may partially account for inter- and intra-patient variability. Six patients presented with concomitant conditions, such as psychotic behaviour or knee pain, that have compromised their endurance tests. As a severity score is lacking in alpha-mannosidosis, patients’ disease burden was evaluated at baseline based on the CHAQ disability index (DI). The patient population included in the rhLAMAN10 study scored differently, ranging from severe to mild disability. A *post-hoc* analysis revealed how, mean changes from baseline to LO showed an improvement in all baseline CHAQ-DI score groups in serum oligosaccharides, 3MWT, 6MWT and percentage of predicted FVC. As of today based on the currently available data, no baseline characteristic can be considered a predictive factor for VA treatment outcome. A post-marketing registry study will help in broadening the understanding of the heterogeneity of the alpha-mannosidosis population and the response to treatment.

The 3MSCT was chosen as an advanced activities-of-daily-living measure as it causes greater stress to the musculoskeletal and cardiorespiratory system, and requires a greater range of motion and muscle strength, compared with level walking (Nightingale et al 2014). A clear improvement in 3MSCT was evident in the paediatric population, which is notable given the progressive physical deterioration typically experienced by patients with AM and provides evidence supporting the effect of velmanase alfa treatment. In some patients, improved 3MSCT was also associated with a decreased reliance on wheelchair use and other walking help or aids.

For many endpoints in this study, the observed improvements were most marked in the paediatric population. To determine whether the 3MSCT improvements in the paediatric population were driven by growth alone, exploratory analyses were conducted within the paediatric age groups of <12 years and 12–17 years (representative of age groups characterised in healthy children by a slower growth and performance development in childhood vs the pubertal growth typical of adolescence). Interestingly, mean improvements in 3MSCT were similar between the two age groups at LO: +10.6 steps/min (+28.5%) in patients <12 years and + 10.7 steps/min (+18.3%) in patients aged 12–17 years, and thus not proportional to the improvement rate expected by growth alone.

The positive results for the 6MWT are consistent with the results from the 3MSCT and also suggest a mobility benefit associated with velmanase alfa treatment. A greater increase in FVC (% predicted) observed in the paediatric is also notable given the poorer pulmonary function at baseline in paediatric patients (mean FVC [%] at baseline 79.6% vs 92.5% in paediatric and adult patients, respectively). The comparative improvements in motor and pulmonary function in those who start treatment as paediatric patients, compared with those who begin treatment as adults, suggest that patients may benefit more from treatment started early in the disease course. This observation is in line with previous studies of long-term outcomes of patients receiving IV ERT for lysosomal storage disorders, and supports that treatment of patients with such disorders is recommended and should start early, preferably pre-symptomatically, to obtain better long-term outcomes (Muenzer 2014; Gabrielli et al 2010; McGill et al 2010; Tylki-Szymanska et al 2012; Tajima et al 2013).

A reduction in serum oligosaccharides and an increase in IgG levels were also achieved in adults. Clinically, these data are highly relevant when considering the vulnerability of this patient population to infections that can cause significant morbidity. In addition, although the magnitude of the treatment effect in domains such as endurance was smaller in adults compared with paediatric patients, the observed level of improvement or stabilisation in adults across all domains is still clinically important because of the progressive nature of the underlying disease. Preservation of adequate pulmonary function and maintenance of endurance and mobility in adult subjects may lead to a better health-related quality of life than would be associated with the gradual deterioration typical of AM.

Velmanase alfa was generally well tolerated throughout the study. A conservative threshold of 1.4 U/ml (lower limit of detection of the assay) was set with regard to detecting ADAs. A limited number of patients developed ADAs, with no clear effect on the co-primary efficacy outcomes (see also Borgwardt et al 2017), despite for one of the two patients with ADA > 80 U/ml who presented oligosaccharides above baseline levels at LO. Three patients experienced IRRs, all of which were mild or moderate in intensity and which resolved either spontaneously or with medical management. Of note, two of these patients had high levels of ADAs (>80 U/ml). A clear correlation between ADA and IRR occurrence has never been established in other therapies. Interestingly, the two patients with highest titres of ADA in VA clinical development programme developed IRR, suggesting the importance of keeping ADA monitoring in the future. The limited number of patients with high titre of ADA does not allow driving conclusions for today and the future registry study will help in providing new insights. The data were collected longitudinally, and form the largest clinical dataset evaluating ERT in AM to date. Our findings suggest that the significant improvements in biochemical and functional efficacy measures associated with velmanase alfa may persist for up to 4 years in paediatric patients, with adult patients experiencing significant improvements in serum oligosaccharide levels and stabilisation of functional performance up to 2 years after treatment initiation. The long-term safety outcomes suggest that there are no additional risks associated with extended treatment. The lack of effect on CSF biomarkers is an area of unmet need and a future research focus.

## Electronic supplementary material

ESM 1 (DOCX 24 kb)

ESM 2 (DOCX 13 kb)

ESM 3 (DOCX 13 kb)

ESM 4 (DOCX 13 kb)

ESM 5 (DOCX 13 kb)

ESM 6 (DOCX 14 kb)

ESM 7 (DOCX 16 kb)

ESM 8 (DOCX 18 kb)

ESM 9 (JPEG 983 kb)

ESM 10 (JPEG 847 kb)
