# Supplementary material for: Comprehensive long-term efficacy and safety of recombinant human alpha-mannosidase (velmanase alfa) treatment in patients with alpha-mannosidosis
Source: J Inherit Metab Dis. 2018 May 3;41(6):1225–33. doi: 10.1007/s10545-018-0175-2 (PMC6326957; doi:10.1007/s10545-018-0175-2)
Supplement: Supplementary file 1 — (DOCX 24 kb) [file 10545_2018_175_MOESM1_ESM.docx]

**Supplementary Table 1** Baseline patient characteristics

|  | **COHORT** | **Sex** | **Age class at 1st dose** | **Genotype** | **Oligosaccharides (umol/L)** | **3MSCT(step/min)** | **6MWT (m)** | **FVC(%of predicted)** |
| --- | --- | --- | --- | --- | --- | --- | --- | --- |
| 30 | rhLAMAN-02/03/04 | F | 12 \|-18 years | Genotype Group 3 | 6 | 62.7 | 454 | 99 |
| 44 | rhLAMAN-02/03/04 | M | 12 \|-18 years | Genotype Group 2 | 9 | 58.3 | 524 | 80 |
| 32 | rhLAMAN-02/03/04 | M | 12 \|-18 years | Genotype Group 2 | 7 | 55.0 | 448 | 92 |
| 27 | rhLAMAN-02/03/04 | M | <12 years | Genotype Group 3 | 8 | 55.0 | 481 | 84.9 |
| 64 | rhLAMAN-02/03/04 | F | <12 years | Genotype Group 2 | 7 | 60.7 | 521 | 95 |
| 36 | rhLAMAN-02/03/04 | M | <12 years | Genotype Group 2 | 10 | 16.7 | 180 | 63.6 |
| 48 | rhLAMAN-02/03/04 | M | 12 \|-18 years | Genotype Group 2 | 11 | 60.7 | 494.5 | 91 |
| 59 | rhLAMAN-02/03/04 | M | 12 \|-18 years | Genotype Group 2 | 15 | 57.7 | 454 | 61 |
| 66 | rhLAMAN-02/03/04 | M | <12 years | Genotype Group 3 | 8 | 47.0 | 519 | 69 |
| 58 | rhLAMAN-05 Active | F | >= 18 years | Genotype Group 1 | 7.5 | 45.3 | 430 | 96 |
| 38 | rhLAMAN-05 Active | M | >= 18 years | Genotype Group 2 | 7 | 45.0 | 422 | 97 |
| 72 | rhLAMAN-05 Active | M | >= 18 years | Genotype Group 1 | 5 | 56.7 | 490 |  |
| 42 | rhLAMAN-05 Placebo | F | >= 18 years | Genotype Group 3 | 4 | 39.7 | 376 | 100 |
| 84 | rhLAMAN-05 Placebo | M | >= 18 years | Genotype Group 3 | 7.3 | 66.7 | 551 | 98 |
| 70 | rhLAMAN-05 Active | M | >= 18 years | Genotype Group 2 | 7.8 | 50.0 | 480 | 119 |
| 94 | rhLAMAN-05 Active | F | >= 18 years | Genotype Group 2 | 5.6 | 48.0 | 452 | 75 |
| 87 | rhLAMAN-05 Active | F | >= 18 years | Genotype Group 2 | 6.1 | 37.7 | 335 | 72 |
| 75 | rhLAMAN-05 Placebo | F | >= 18 years | Genotype Group 2 | 5.1 | 45.7 | 449 | 51 |
| 91 | rhLAMAN-05 Active | M | >= 18 years | Genotype Group 3 | 6.4 | 47.3 | 491 |  |
| 79 | rhLAMAN-05 Placebo | M | >= 18 years | Genotype Group 2 | 2.3 | 71.3 | 690 | 114 |
| 50 | rhLAMAN-05 Active | M | >= 18 years | Genotype Group 2 | 4.9 | 70.3 | 627 | 103 |
| 46 | rhLAMAN-05 Active | F | >= 18 years | Genotype Group 1 | 7.1 | 48.0 | 422 | 80 |
| 89 | rhLAMAN-05 Placebo | F | >= 18 years | Genotype Group 1 | 6.6 | 70.3 | 552 | 105 |
| 61 | rhLAMAN-05 Placebo | F | 12 \|-18 years | Genotype Group 3 | 4.6 | 37.7 | 408 | 99 |
| 78 | rhLAMAN-05 Placebo | F | 12 \|-18 years | Genotype Group 2 | 4.6 | 65.3 | 537 | 83 |
| 80 | rhLAMAN-05 Active | M | 12 \|-18 years | Genotype Group 1 | 7.9 | 45.3 | 480 | 76 |
| 99 | rhLAMAN-05 Active | M | 12 \|-18 years | Genotype Group 2 | 6 | 83.3 | 586 | 94 |
| 56 | rhLAMAN-05 Active | F | <12 years | Genotype Group 1 | 8.1 | 55.0 | 434 | 50 |
| 33 | rhLAMAN-05 Active | M | <12 years | Genotype Group 2 | 5.9 | 55.0 | 420 | 54 |
| 85 | rhLAMAN-05 Placebo | M | 12 \|-18 years | Genotype Group 2 | 5.7 | 62.7 | 501 | 97 |
| 51 | rhLAMAN-05 Active | M | <12 years | Genotype Group 2 | 8.7 | 53.7 | 427 | 64 |
| 90 | rhLAMAN-05 Placebo | M | <12 years | Genotype Group 1 | 4.8 | 41.7 | 364 |  |
| 37 | rhLAMAN-05 Active | F | <12 years | Genotype Group 2 | 7.7 | 53.3 | 398 |  |
|  |  |  |  |  |  |  |  |  |

Genotype Group 1: Two null-mutations (nonsense, frameshift, large truncation). Subcellular localisation of the mutant mannosidase alpha class 2B member 1 (MAN2B1) proteins was not studied. Genotype Group 2: At least one missense mutation (or in-frame deletion/duplication of 1-5 amino acids) with the MAN2B1 protein localised to the endoplasmic reticulum (ER) (i.e.: ER/ER, ER/null). Genotype Group 3: At least one missense mutation (or in-frame deletion/duplication of 1-5 amino acids) with the MAN2B1 protein localised to the lysosomes (i.e.: lyso/lyso, lyso/ER, lyso/null)
