# Supplementary material for: Comprehensive long-term efficacy and safety of recombinant human alpha-mannosidase (velmanase alfa) treatment in patients with alpha-mannosidosis
Source: J Inherit Metab Dis. 2018 May 3;41(6):1225–33. doi: 10.1007/s10545-018-0175-2 (PMC6326957; doi:10.1007/s10545-018-0175-2)
Supplement: Supplementary file 2 — (DOCX 13 kb) [file 10545_2018_175_MOESM2_ESM.docx]

**Supplementary Table 2** Pure Tone Audiometry

|  | | **Baseline** | **Change from baseline**  **to last observation** | |
| --- | --- | --- | --- | --- |
|  |  |  | **Absolute** | **%** |
| **Bone conduction (best ear)** | | | | |
| **Overall** | ***n*** | 32 | 32 | 32 |
|  | **Mean (SD)** | 52.6 (12.4) | –0.49 (6.58) | –0.72 (14.5) |
| **Paediatric** | ***n*** | 18 | 18 | 18 |
|  | **Mean (SD)** | 47.9 (11.9) | –2.04 (7.39) | –4.03 (17.1) |
| **Adult** | ***n*** | 14 | 14 | 14 |
|  | **Mean (SD)** | 58.6 (10.5) | 1.49 (4.92) | 3.54 (9.27) |
| **Air conduction (left ear)** | | | | |
| **Overall** | ***n*** | 33 | 33 | 33 |
|  | **Mean (SD)** | 62.3 (16.5) | –2.83 (7.14) | –3.79 (10.7) |
| **Paediatric** | ***n*** | 19 | 19 | 19 |
|  | **Mean (SD)** | 60.5 (19.1) | –4.15 (8.52) | –5.68 (12.4) |
| **Adult** | ***n*** | 14 | 14 | 14 |
|  | **Mean (SD)** | 64.9 (12.4) | –1.03 (4.37) | –1.23 (7.56) |
| **Air conduction (right ear)** | | | | |
| **Overall** | ***n*** | 33 | 33 | 33 |
|  | **Mean (SD)** | 61.9 (17.9) | –1.41 (10.3) | 0.54 (20.2) |
| **Paediatric** | ***n*** | 19 | 19 | 19 |
|  | **Mean (SD)** | 57.1 (18.3) | –0.43 (10.6) | 3.32 (24.6) |
| **Adult** | ***n*** | 14 | 14 | 14 |
|  | **Mean (SD)** | 68.2 (15.9) | –2.74 (10.1) | –3.23 (11.9) |
