# Supplementary material for: Comprehensive long-term efficacy and safety of recombinant human alpha-mannosidase (velmanase alfa) treatment in patients with alpha-mannosidosis
Source: J Inherit Metab Dis. 2018 May 3;41(6):1225–33. doi: 10.1007/s10545-018-0175-2 (PMC6326957; doi:10.1007/s10545-018-0175-2)
Supplement: Supplementary file 3 — (DOCX 13 kb) [file 10545_2018_175_MOESM3_ESM.docx]

|  | | **Baseline** | **Change from baseline**  **to last observation** | |
| --- | --- | --- | --- | --- |
|  |  |  | **Absolute** | **%** |
| **Overall** | ***n*** | 33 | 33 | 33 |
|  | **Mean (SD)** | 5.88 (1.57) | 0.27 (0.64) | 5.34 (10.5) |
| **Paediatric** | ***n*** | 19 | 19 | 19 |
|  | **Mean (SD)** | 5.40 (1.40) | 0.32 (0.52) | 6.75 (9.82) |
| **Adult** | ***n*** | 14 | 14 | 14 |
|  | **Mean (SD)** | 6.53 (1.59) | 0.20 (0.79) | 3.42 (11.3) |

**Supplementary Table 3** Leiter-R total equivalent age (visualisation and reasoning) (years) at baseline and last observation overall, and by age
