# Supplementary material for: Comprehensive long-term efficacy and safety of recombinant human alpha-mannosidase (velmanase alfa) treatment in patients with alpha-mannosidosis
Source: J Inherit Metab Dis. 2018 May 3;41(6):1225–33. doi: 10.1007/s10545-018-0175-2 (PMC6326957; doi:10.1007/s10545-018-0175-2)
Supplement: Supplementary file 4 — (DOCX 13 kb) [file 10545_2018_175_MOESM4_ESM.docx]

**Supplementary Table 4** MRI/MRS imaging: Apparent diffusion coefficient (ADC)

Imaging was only carried out on patients who participated in the Phase I trial.

|  | **Mean absolute change from baseline (*P* value; 95% CI)** | **Mean % change from baseline (*P* value; 95% CI)** |
| --- | --- | --- |
| **ADC (grey)** |  |  |
| *n* | 9 | 9 |
| Month 12 | –32.0  (0.032; –60.4, –3.6) | –3.9  (0.030; –7.3, –0.5) |
| Last observation | 160.2  (< 0.001; 108, 213) | 20.3  (< 0.001; 13.5, 27.0) |
| **ADC (standard)** |  |  |
| *n* | 9 | 9 |
| Month 12 | –9.6  (0.443; –36.8, 17.7) | –1.0  (0.491; –4.2, 2.2) |
| Last observation | –5.7  (0.699; –38.3, 27.0) | –0.3  (0.830; –3.8, 3.2) |
| **ADC (white)** |  |  |
| *n* | 9 | 9 |
| Month 12 | 33.4  (0.184; –19.6, 86.5) | 4.4  (0.160; –2.1, 10.9) |
| Last observation | –113  (0.007; –185, –40.8) | –11.5  (0.003; –17.8, –5.1) |
