# Supplementary material for: Comprehensive long-term efficacy and safety of recombinant human alpha-mannosidase (velmanase alfa) treatment in patients with alpha-mannosidosis
Source: J Inherit Metab Dis. 2018 May 3;41(6):1225–33. doi: 10.1007/s10545-018-0175-2 (PMC6326957; doi:10.1007/s10545-018-0175-2)
Supplement: Supplementary file 5 — (DOCX 13 kb) [file 10545_2018_175_MOESM5_ESM.docx]

**Supplementary Table 5** Mannose complex visual

|  | **Mean absolute change from baseline (*P* value; 95% CI)** | **Mean % change from baseline (*P* value; 95% CI)** |
| --- | --- | --- |
| **Mannose complex visual (grey)** |  |  |
| *n* | 9 | 9 |
| Month 12 | –0.3  (0.081; –0.7, 0.1) | –16.7  (0.081; –35.9, 2.6) |
| Last observation | –0.4  (0.035; –0.8, 0.0) | –33.3  (0.050; –66.6, 0.0) |
| **Mannose complex visual (standard)** |  |  |
| *n* | 8 | 6 |
| Month 12 | 0.0  (N/A) | 0.0  (N/A) |
| Last observation | –0.1  (0.598; –0.7, 0.4) | –8.3  (0.611; –47.8, 31.2) |
| **Mannose complex visual (white)** |  |  |
| *n* | 9 | 7 |
| Month 12 | –0.3  (0.195; –0.9, 0.2) | –21.4  (0.200; –57.8, 15.0) |
| Last observation | 0.0  (1.000; –0.7, 0.7) | –4.8  (0.815; –52.4, 42.9) |
