# Supplementary material for: Comprehensive long-term efficacy and safety of recombinant human alpha-mannosidase (velmanase alfa) treatment in patients with alpha-mannosidosis
Source: J Inherit Metab Dis. 2018 May 3;41(6):1225–33. doi: 10.1007/s10545-018-0175-2 (PMC6326957; doi:10.1007/s10545-018-0175-2)
Supplement: Supplementary file 6 — (DOCX 14 kb) [file 10545_2018_175_MOESM6_ESM.docx]

**Supplementary Table 6** CSF oligosaccharides at baseline and last observation overall, and by age

|  | | **Baseline** | **Change from baseline**  **to last observation** | |
| --- | --- | --- | --- | --- |
|  |  |  | **Absolute** | **%** |
| **CSF oligosaccharides** | | | | |
| **Overall** | ***n*** | 33 | 33 | 33 |
|  | **Mean**  **(SD)** | 10.6  (3.53) | –0.59  (1.97) | –3.54  (20.8) |
| **Paediatric** | ***n*** | 19 | 19 | 19 |
|  | **Mean**  **(SD)** | 10.7  (3.84) | –0.78  (2.33) | –5.44  (22.4) |
| **Adult** | ***n*** | 14 | 14 | 14 |
|  | **Mean**  **(SD)** | 10.6  (3.20) | –0.33  (1.38) | –0.97  (18.9) |
| **Tau protein** | | | | |
| **Overall** | ***n*** | 33 | 33 | 33 |
|  | **Mean**  **(SD)** | 656  (318) | 20.8  (163) | 7.7  (26.7) |
| **Paediatric** | ***n*** | 19 | 19 | 19 |
|  | **Mean**  **(SD)** | 722  (338) | 15.5  (192) | 3.7  (24.7) |
| **Adult** | ***n*** | 14 | 14 | 14 |
|  | **Mean**  **(SD)** | 566  (277) | 28.0  (119) | 13.2  (29.3) |
| **Neurofilament protein** | | | | |
| **Overall** | ***n*** | 33 | 33 | 33 |
|  | **Mean**  **(SD)** | 430  (256) | –26.2  (209) | 11.6  (46.1) |
| **Paediatric** | ***n*** | 19 | 19 | 19 |
|  | **Mean**  **(SD)** | 436  (248) | –32.6  (228) | 13.6  (56.0) |
| **Adult** | ***n*** | 14 | 14 | 14 |
|  | **Mean**  **(SD)** | 422  (276) | –17.5  (189) | 8.9  (29.7) |
| **Glial fibrillary acidic protein** | | | | |
| **Overall** | ***n*** | 33 | 33 | 33 |
|  | **Mean**  **(SD)** | 521  (379) | 175  (499) | 102  (148) |
| **Paediatric** | ***n*** | 19 | 19 | 19 |
|  | **Mean**  **(SD)** | 575  (435) | 12.6  (516) | 86.3  (172) |
| **Adult** | ***n*** | 14 | 14 | 14 |
|  | **Mean**  **(SD)** | 447  (285) | 395  (391) | 123  (110) |
