# Supplementary material for: Comprehensive long-term efficacy and safety of recombinant human alpha-mannosidase (velmanase alfa) treatment in patients with alpha-mannosidosis
Source: J Inherit Metab Dis. 2018 May 3;41(6):1225–33. doi: 10.1007/s10545-018-0175-2 (PMC6326957; doi:10.1007/s10545-018-0175-2)
Supplement: Supplementary file 7 — (DOCX 16 kb) [file 10545_2018_175_MOESM7_ESM.docx]

**Supplementary Table 7** PK parameters for patients who received velmanase alfa at 25 U/kg or 1 mg/kg dose level (PK analysis set)

|  |  | **Day 1*** | **Steady state** |
| --- | --- | --- | --- |
| **AUC_0–t_ (h*μg/l)** | *n* | 16 | 12 |
|  | Mean (SD) | 87052.51 (27110.75) | 143128.85 (32730.06) |
|  | Median  (min, max) | 84924.41  (40480.54, 134454.42) | 141186.35  (91882.00, 202998.52) |
|  | CV% | 31.14 | 22.87 |
|  | Geometric mean | 82771.20 | 139614.45 |
| **AUC_0–∞_ (h*μg/l)** | *n* | 15 | 12 |
|  | Mean (SD) | 99913.60 (27792.01) | 158877.64 (38824.62) |
|  | Median  (min, max) | 97966.78  (46911.37, 145172.35) | 155211.96  (98340.26, 228700.71) |
|  | CV% | 27.82 | 24.44 |
|  | Geometric mean | 95956.32 | 154395.38 |
| **%AUC_extr_ (%)** | *n* | 15 | 12 |
|  | Mean (SD) | 10.42 (2.89) | 9.54 (2.48) |
|  | Median  (min, max) | 10.28  (6.98,18.50) | 8.91  (6.57, 16.00) |
|  | CV% | 27.77 | 26.04 |
|  | Geometric mean | 10.10 | 9.29 |
| **R_ac_ (%)** | *n* |  | 12 |
|  | Mean (SD) |  | 1.589 (0.257) |
|  | Median  (min, max) |  | 1.621  (0.996, 1.942) |
|  | CV% |  | 16.162 |
|  | Geometric mean |  | 1.567 |
| **C_max_ (μg/l)** | *n* | 16 | 12 |
|  | Mean (SD) | 8488.75 (4463.84) | 7485.00 (1100.33) |
|  | Median  (min, max) | 7775.00  (4000.00, 23200.00) | 7555.00  (5570.00, 9650.00) |
|  | CV% | 52.59 | 14.70 |
|  | Geometric mean | 7729.78 | 7409.39 |
| **t_max_ (h)** | *n* | 16 | 12 |
|  | Mean (SD) | 1.851 (0.931) | 1.781 (0.317) |
|  | Median  (min, max) | 1.750  (0.667, 3.500) | 1.658  (1.483, 2.483) |
|  | CV% | 50.294 | 17.777 |
|  | Geometric mean^†^ | 1.714 | 1.765 |
| **Cl (L/h/kg)** | *n* | 15 | 12 |
|  | Mean (SD) | 0.0107 (0.0038) | 0.0067 (0.0018) |
|  | Median  (min, max) | 0.0091  (0.0069, 0.0213) | 0.0065  (0.0044, 0.0103) |
|  | CV% | 35.4021 | 26.4465 |
|  | Geometric mean | 0.0102 | 0.0065 |
| **V (L/kg)** | *n* | 15 | 12 |
|  | Mean (SD) | 0.217 (0.037) | 0.274 (0.037) |
|  | Median  (min, max) | 0.216  (0.137,0.280) | 0.267  (0.209, 0.339) |
|  | CV% | 17.130 | 13.479 |
|  | Geometric mean | 0.213 | 0.272 |
| **t_1/2_ (h)** | *n* | 15 | 12 |
|  | Mean (SD) | 15.13 (4.21) | 29.88 (6.88) |
|  | Median  (min, max) | 14.58  (7.91, 21.08) | 31.53  (18.35, 40.93) |
|  | CV% | 27.85 | 23.01 |
|  | Geometric mean | 14.53 | 29.06 |
| **λz (/h)** | *n* | 15 | 12 |
|  | Mean (SD) | 0.0500 (0.0172) | 0.0246 (0.0069) |
|  | Median  (min, max) | 0.0475  (0.0329, 0.0876) | 0.0220  (0.0169, 0.0378) |
|  | CV% | 34.3303 | 27.9022 |
|  | Geometric mean | 0.0477 | 0.0239 |

AUC_0-t_, the area under the plasma concentration curve observed from administration up to the last measurable concentration; AUC_0-∞_, the area under the plasma concentration curve extrapolated to infinity; Cl, apparent total body clearance; C_max_, maximum plasma concentration; CV, coefficient of variation; max, maximum; min, minimum; n, number of patients with available data; R_ac_, accumulation ratio between steady state and day 1 dosing; SD, standard deviation; t_1/2_, terminal half life; t_max_, time to maximum plasma concentration; λz, terminal elimination rate.

* Day 1, PK assessment of first velmanase alfa administration; Steady state, Last PK assessment of rhLAMAN-07/rhLAMAN-09/rhLAMAN-10.

^†^ Geometric mean of t_max_ was approximated by the plus one method because of zero t_max_ values.
