# Supplementary material for: Comprehensive long-term efficacy and safety of recombinant human alpha-mannosidase (velmanase alfa) treatment in patients with alpha-mannosidosis
Source: J Inherit Metab Dis. 2018 May 3;41(6):1225–33. doi: 10.1007/s10545-018-0175-2 (PMC6326957; doi:10.1007/s10545-018-0175-2)
Supplement: Supplementary file 8 — (DOCX 18 kb) [file 10545_2018_175_MOESM8_ESM.docx]

# **Supplementary methods**

## Key inclusion/exclusion criteria for studies included in this analysis

Inclusion/exclusion criteria for the Phase I/II and Phase III trials are published elsewhere (Borgwardt et al 2013; Borgwardt et al 2017)

### Key inclusion/exclusion criteria for rhLAMAN-10

(NCT02478840)

Key inclusion criteria:

- The subject must have participated in the Phase I/II or Phase III trials
- The subject must still be receiving weekly intravenous infusions of velmanase alfa according to the CU programme
- The Subject or subjects legally authorized guardian(s) must provide signed, informed consent prior to performing any trial-related activities
- The subject and his/her guardian(s) must have the ability to comply with the protocol

Exclusion Criteria:

- History of bone marrow transplantation
- Presence of known clinically significant cardiovascular, hepatic, pulmonary or renal disease or other medical conditions that, in the opinion of the Investigator, would preclude participation in the trial. Subjects unable to perform the motor tests independently from support are permitted to participate in the trial and will be evaluated for the remnant non-motor endpoints
- Any other medical condition or serious intercurrent illness, or extenuating circumstance that, in the opinion of the investigator, would preclude participation in the trial
- Pregnant and/or lactating women cannot participate in the trial. Concerning women of child bearing potential (WOCBP), the investigators will decide whether or not there is a need for contraception. This assessment will be done through interviews with the patient and parents.
- Participation in other interventional trials testing investigational medical products, including rhLAMAN-07 and rhLAMAN-09 trials

Key inclusion/exclusion criteria for rhLAMAN-07 and rhLAMAN-09

(NCT01908712; NCT01908725)

Inclusion Criteria:

- The subject must have participated in previous Phase II or Phase III Lamazym trials
- Subject or subjects legally authorized guardian(s) must provide signed, informed consent prior to performing any trial-related activities
- The subject and his/her guardian(s) must have the ability to comply with the protocol

Exclusion Criteria:

- Presence of known clinically significant cardiovascular, hepatic, pulmonary, or renal disease or other medical conditions that, in the opinion of the Investigator, would preclude participation in the trial
- Any other medical condition or serious intercurrent illness, or extenuating circumstances that, in the opinion of the Investigator, would preclude participation in the trial
- Pregnancy. Pregnant women are excluded. Before the start of treatment the investigators will perform a pregnancy test for women of childbearing potential and decide whether or not there is a need for contraception
- Psychosis; any psychotic disease, also in remission, is an exclusion criteria
- Planned major surgery that, in the opinion of the Investigator, would preclude participation in the trial

## Additional details on BOT-2 subtests

The BOT-2 subtest point scores from the Fine Motor Precision (FMP), Fine Motor Integration (FMI), Manual Dexterity (MD), Upper Limb Coordination (ULC), Bilateral Coordination (BC), Balance, and Running Speed and Agility (RSA) subtests were summed to derive a total point score and the individual subtests were evaluated against normative data.
