# Supplementary figures and images for: Comprehensive long-term efficacy and safety of recombinant human alpha-mannosidase (velmanase alfa) treatment in patients with alpha-mannosidosis
Source: J Inherit Metab Dis. 2018 May 3;41(6):1225–33. doi: 10.1007/s10545-018-0175-2 (PMC6326957; doi:10.1007/s10545-018-0175-2)

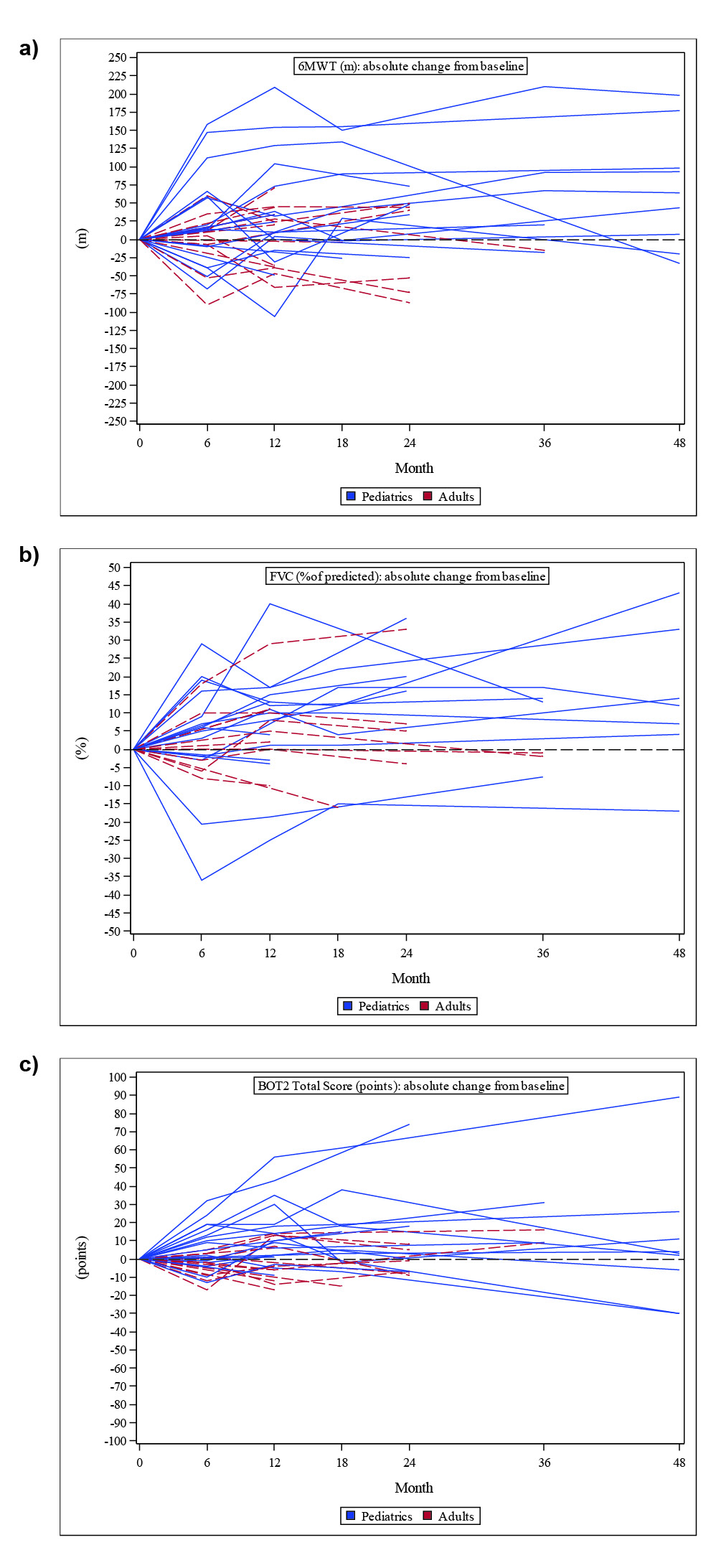

Supplement: Supplementary file 9 — (JPEG 983 kb) [file 10545_2018_175_MOESM9_ESM.jpg]

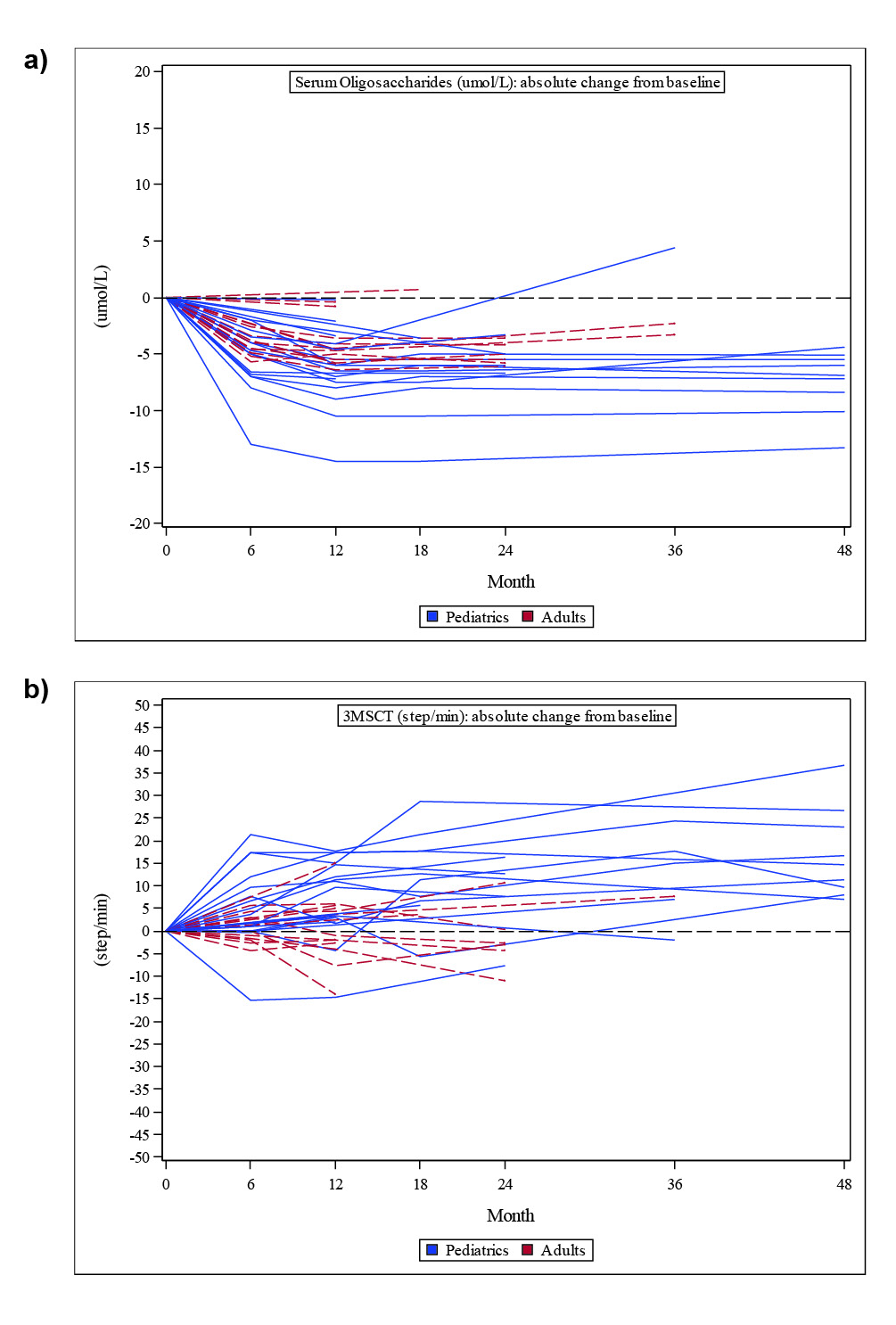

Supplement: Supplementary file 10 — (JPEG 847 kb) [file 10545_2018_175_MOESM10_ESM.jpg]
